# Investigating 3D-Printed Carbon–Carbonyl Iron Composites for Electromagnetic Applications

**DOI:** 10.3390/polym17081009

**Published:** 2025-04-08

**Authors:** Dzmitry Tsyhanok, Darya Meisak, Pauline Blyweert, Algirdas Selskis, Jan Macutkevič, Jūras Banys, Vanessa Fierro, Alain Celzard

**Affiliations:** 1 Physics Faculty, Vilnius University, Sauletekio av. 9, LT-10222 Vilnius, Lithuania; dzmitry.tsyhanok@ff.vu.lt (D.T.); darya.meisak@ff.vu.lt (D.M.); juras.banys@ff.vu.lt (J.B.); 2 CNRS—Centre National de la Recherche Scientifique, Université de Lorraine—IJL, F-88000 Épinal, France; pauline.blyweert@univ-reims.fr (P.B.); vanessa.fierro@univ-lorraine.fr (V.F.); alain.celzard@univ-lorraine.fr (A.C.); 3 Center for Physical Science and Technology, Sauletekio av. 3, LT-10257 Vilnius, Lithuania; algirdas.selskis@ftmc.lt; 4 Institut Universitaire de France (IUF), F-75231 Paris, France

**Keywords:** dielectric permittivity, microwave absorption, 3D-printed structures

## Abstract

The electromagnetic properties of 3D-printed carbon–carbonyl iron powder (CIP) composites are studied in the radio (20 Hz–1 MHz) and microwave (26–37 GHz) frequency ranges. Relatively high electrical conductivities (about several hundred S/m), typical for these structures in the radio frequency range, are observed. The temperature dependence of electrical conductivity is described by Arrhenius’ law, with distinct activation energies above and below a critical temperature, attributed to electron transport through various defects. The microwave properties of the investigated structures are particularly noteworthy. For instance, a 2 mm-plate with 20 wt.% magnetic inclusions achieves 52% absorption at 35 GHz. The microwave dielectric properties of the composite structures strongly depend on the concentration of carbonyl iron particles, with the highest values of the imaginary part of complex dielectric permittivity observed in carbon structures containing 20 wt.% CIP. Moreover, carbon composites with the highest CIP concentration exhibited interesting resonance states, demonstrating significant potential for Salisbury screen applications.

## 1. Introduction

The use of electromagnetic waves in many aspects of modern life is increasing rapidly. However, this growth has intensified the issue of electromagnetic interference, particularly as more devices operate in the same frequency range. The problem is further exacerbated by the rapid increase in the power and frequency of electromagnetic radiation, which is required to transmit greater quantities of information. Electromagnetic interference can cause the damages of electronic devices and can be harmful for human health. Therefore, the development and improvement of advanced materials and technologies for electromagnetic shielding and absorption have become critical priorities.

Various materials have been investigated for electromagnetic interference shielding applications. Magnetic (ferromagnetic, antiferromagnetic and others) materials are popular in such applications due to their relatively high complex permeability (µ* = µ′ + µ″) [1]. However, the real part of permeability exhibits an abrupt decrease at microwave frequencies at the same time the imaginary part presents a resonance, i.e. a Snoek limit is observed; this phenomenon seriously limits applications of magnetic materials [2]. Carbonyl iron powder (CIP) is a popular filler used in various composites designed for electromagnetic shielding applications [3]. Its affordability, high-specification saturation and favorable Snoeck limit make it particularly valuable for microwave applications [4]. Moreover, experimental investigations showed that the real part of magnetic permeability of CIP particles in microwaves increase with particle size [5]. Notably, the electromagnetic properties of CIP composites with polymeric matrices such as polychloroprene, polyurethane, polyaniline, epoxy resin and polyvinylidene fluoride, containing relatively high CIP concentrations (up to 95%), have been extensively studied in different frequency ranges for microwave shielding applications [6,7,8,9,10]. These composites demonstrate excellent magnetic and dielectric properties in the microwave frequency range, but their electrical conductivity remains relatively low.

Another class of materials commonly used for electromagnetic shielding applications includes various carbon allotropes, such as carbon nanotubes (CNT), carbon black (CB), graphite, graphene and porous carbon [11,12]. These insulator–conductor composites at some critical concentration (called electrical percolation threshold) become electrically conductive. Moreover, composites with CNT exhibit ultralow electrical percolation threshold values due to high aspect ratio of these fillers, for example, in epoxy resin filled with multi-wall CNT (MWCNT), the percolation threshold was reported as 0.0025 wt.% [13], in ultrahigh-molecular-weight polyethylene, the percolation threshold was established as 0.095 wt.% for single-wall CNT (SWCNT) and 0.05 wt.% for MWCNT [14]. The systems with other carbon allotropes can also exhibit very low percolation threshold values, for example, in epoxy resin composites filled with onion-like carbon (OLC) the percolation threshold can be 0.9 vol.% [15], in composites with graphene inclusions, the percolation threshold can be close to 0.5 vol.% [16]. Recently, carbon foams, gels and spheres have been proposed as materials for electromagnetic applications [17,18,19,20]. Studies have shown that the density of carbon foams and gels is a key parameter determining their microwave absorption properties [17]. However, it was also demonstrated that the morphological control of carbon network structures play a crucial role on their electromagnetic properties [18]. Such control of carbon network structures can be performed by the use various technological methods, including shear, polymer blends, thermal annealing and mixed filler [18]. It was demonstrated that hollow carbon spheres may be used for building an extremely lightweight, almost perfectly absorbing, coating for 26–37 GHz frequency range applications [20]. Composites incorporating CNTs, CB and other carbon-based nano-inclusions exhibit rather high electrical conductivity and effective microwave shielding ability at filler concentrations well above the percolation threshold [21]. Above the percolation threshold, the electrical conductivity increases in a power law fashion and can reach high electrical conductivity values enough for electromagnetic shielding applications. For example, electromagnetic shielding of composites with SWCNT can reach 46 dB at 3 GHz frequency and filler concentration 3 wt.% [21], electromagnetic shielding of composites with MWCNT can reach 80 dB at 12.5 GHz frequency and filler concentration 5.5 vol.% [22], while electromagnetic shielding of composites with graphene can reach 90 dB at 12 GHz frequency and filler concentration 5.5 vol.% [23]. The percolation threshold can be very low in such composites with proper filler distribution within the polymer matrix [24,25]. However, a major drawback of these composites is their relatively high electrical conductivity, which results in greater microwave reflection than absorption [26]. Another challenge with the use of carbon nanoparticles as fillers is their tendency to form agglomerates in the polymeric matrix. As a result, the electromagnetic properties of these composites are primarily governed by the conductivity of the clusters and their distribution in the matrix [25].

Efficient electromagnetic shielding requires specific ranges of complex dielectric permittivity and magnetic permeability. The modification of carbon-based materials by the addition of magnetically or ferroelectrically active particles is also a widely adopted strategy [27,28,29]. Particularly, in the work [27], reduced graphene oxide–Fe aerogel composites were prepared and demonstrated that their electromagnetic properties can be tailored by varying the concentrations of Fe^2−^ ions. However, comprehensive studies of carbon-based, broadband electromagnetic materials with ferromagnetic inclusions are still lacking. In this work, we investigated the effect of incorporating CIP into different 3D-printed carbon structures on their dielectric properties and demonstrate that these structures are suitable for electromagnetic applications.

## 2. Materials and Methods

Carbon–carbonyl iron composite samples were prepared using a methodology combining 3D printing of a bio-based composite resin followed by heat treatment [29]. Briefly, a photocurable acrylate-tannin-carbonyl iron resin was obtained by first mixing the photoinitiator BAPO (phenylbis(2,4,6-trimethylbenzoyl)phosphine oxide, 0.3 wt.%, supplied by Lambson, Wetherby, England) with aromatic acrylate oligomer CN154 CG, aliphatic pentaerythritol tetraacrylate (PETA, SR295) and 1,6-hexanediol diacrylate (HDDA, SR238), supplied by Sartomer (Arkema Group, Verneuil en Halatte, France) in a 4:4:2 ratio. Subsequently, tannin (25 wt.%) and carbonyl iron particles (purity > 98%, spherical particles between 1 and 3 µm, Alfa Aesar, Haverhill, MA, USA), Strasbourg, France in weight fractions Φ**_i_** = 0, 5, 10 or 15 wt.%, were added and stirred for a few minutes to ensure good dispersion in the resin. An initial particle concentration above 15 wt.% significantly increases light adsorption by the resin, which hampers the laser stereography printing process (see below), and tends to reduce the mechanical strength of the printed part.

The resultant resins were processed in a DWS J28 desktop SLA (405 nm laser, DWS, Venice, Italy) to print cylinders (6.3 mm diameter and 8 mm height) and rods (1.33 mm diameter and 15.10 mm height, to target waveguide dimensions after shrinkage induced during pyrolysis). The printed structures were post-cured in a UV oven (405 nm) for 20 min at room temperature and finally converted into highly disordered carbon materials under pure nitrogen (flow rate 75 mL/min) in a tubular furnace with a heating ramp of 1 °C/min to a final temperature of 900 °C, which was maintained for 1 h. The main properties of the carbon materials obtained by this method have been detailed elsewhere [30,31,32].

Scanning electron microscopy (SEM) images were obtained using a JSM 6460 LV electron microscope, JEOL, Tokyo, Japan. During heat treatment, the photosensitive resin completely decomposed, and the tannin decomposed by around 55%, giving a final carbon yield of 20.8% of its original mass. The CIP mass remained constant. As a result, samples with a final CIP content (Φ_f_) of 0, 20, 35 and 46 wt.% dispersed within an amorphous and porous carbon matrix were obtained.

It should be noted that, due to the high stresses experienced during pyrolysis, long and thin materials could have undergone slight deformation. However, it was always possible to obtain straight parts suitable for the waveguide (approximately 3.5 mm in height). Linear shrinkage was around 25% in all directions, with no observable influence from the presence of CIP particles.

Due to the high density of the carbonyl iron powder (7.86 g/cm^3^), increasing the CIP content in the photosensitive resin resulted in composites with progressively higher densities ranging from 0.42 to 0.67 g/cm^3^ (details are provided in Table 1). Consequently, the resulting materials had low volume fractions of magnetic particles.

**Figure 1:**
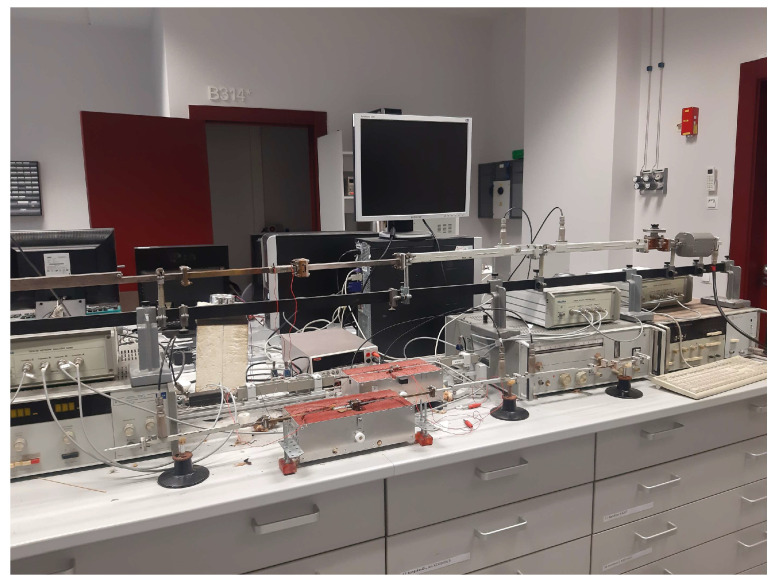
Setup for microwave dielectric measurements.

In the frequency range 20 Hz–1 MHz, electrical properties were measured using a Hewlett-Packard 4284A LCR-meter, Spring, TX, USA. The complex impedance was measured using the method of equivalent circuits. The electrical conductivity was calculated according to the formula σ = l/(S∙R), where l and S are the length and the cross-sectional area of the sample, respectively, and R is the measured electrical resistance. The electrical conductivity was also investigated in the temperature range 120–300 K with a liquid nitrogen cryostat. Silver paint was used to make electrical contacts. The dielectric properties of the structures were also investigated in the microwave frequency range (26–38 GHz, Ka-band) using a 7.2 × 3.4 mm^2^ rectangular cross-section waveguide (Figure 1). An Elmika 2400 scalar network analyzer, Elmika, Vilnius, Lithuania was used for scattering parameters measurements. Rod-like samples with a diameter of about 1 mm and a length of about 3.5 mm were investigated and placed in the center of the waveguide with their axis parallel to the electric field vector. All samples were fixed to the sample holder with silver paint. The complex dielectric permittivity was calculated using a modified Newton optimization algorithm based on the microwave theory formalism (see [33] for details). This method is in good agreement with other microwave dielectric permittivity determination methods [33]. The measurement accuracy was ~10%.

## 3. Results

**Figure 2:**
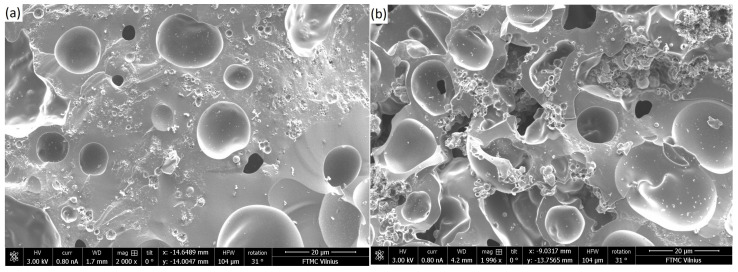
Scanning electron microscopy images of fractured carbon–CIP composites with (**a**) 35 wt.% and (**b**) 46 wt.% CIP particles.

SEM images of carbon structures with different CIP concentrations, shown in Figure 2, revealed a rather homogeneous dispersion of the CIP particles in the carbon matrix. The quality of dispersion of CIP nanoparticles in carbon matrix is independent from their concentration.

**Figure 3:**
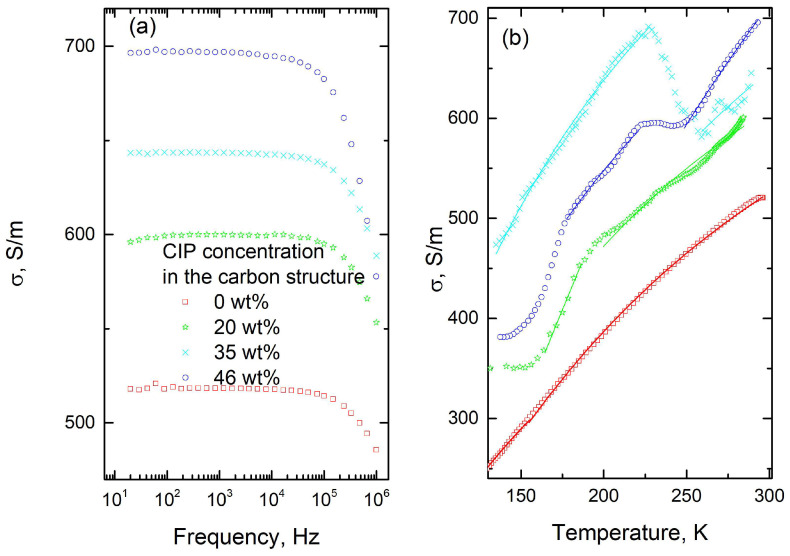
(**a**) Electrical conductivity spectra in the 20 Hz–1 MHz frequency range of carbon structures with different wt.% CIP. (**b**) Temperature dependence of DC conductivity for the same structures. Solid lines are the best fits according to Arrhenius’ law (Equation (2)).

Figure 3a presents the frequency dependencies of the electrical conductivity of carbon and carbon–CIP structures at room temperature. Electrical conductivity is only slightly dependent on frequency, suggesting that it approximates direct electrical conductivity (DC conductivity).

The electrical resistivity of carbonyl iron is 9.71 µΩ·cm [34,35], so its conductivity (10.3 × 10^6^ S/m), is significantly higher than that of the carbon structure without magnetic additions (518 S/m). According to the Maxwell–Garnett theory, electrical conductivity is expected to increase with CIP concentration [10]. Composite density also plays a critical role, with electrical conductivity generally increasing with density according to the following power law [17]:

σ~d^s^, (1)

where d (dimensionless) is the relative density, and s is a parameter dependent on the composite structure. Other factors that can also affect the electrical conductivity of structures can be the creation of various charge carriers (including electrons and holes) during pyrolysis of the polymer structure. Additionally, we can admit that the concentration of CIP is relatively low (up to 4 vol.%); therefore, it can be expected that the composites were below the percolation threshold and that the electrical conductivity changed only a little with filler concentration [36].

The composite materials studied exhibit typical semiconducting behavior, with electrical conductivity increasing with temperature (Figure 3b). The temperature dependence of electrical conductivity was approximated with Arrhenius’ law separately, below and above a distinct critical temperature (solid lines in Figure 3b):

(2) $$\sigma =\sigma_{0}e^{-\frac{E}{kT}}$$

where σ_0_ is the pre-exponential factor, E is the activation energy and k is the Boltzmann constant.

The approximation parameters are presented in Table 2. Activation energy values are of the order of several tens of meV, similar to those observed in carbon gels or carbon structures filled with BaTiO_3_ [18,29,32]. This suggests that electrical conductivity can be attributed primarily to electron transport within the carbon matrix through various defects.

**Figure 4:**
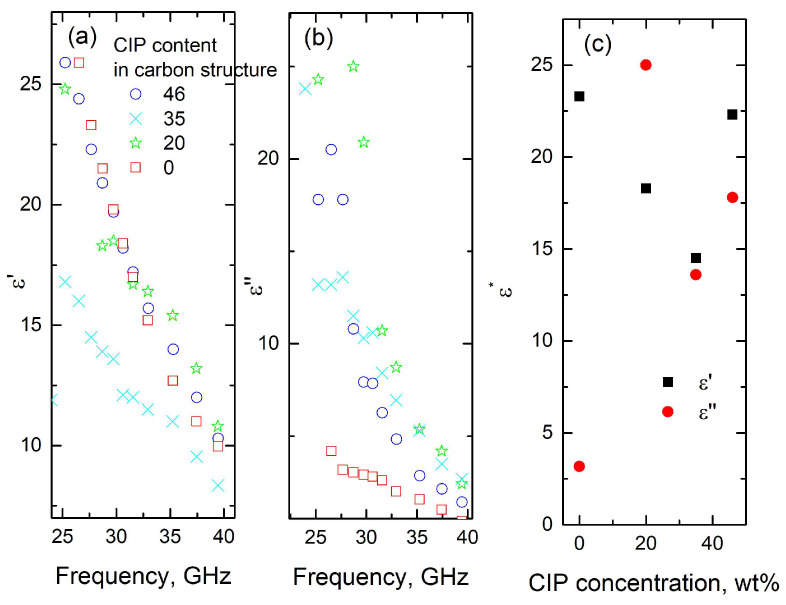
Frequency dependence of carbon–CIP composites: (**a**) dielectric permittivity ε′; (**b**) dielectric losses ε″; (**c**) complex dielectric permittivity of the composites as a function of CIP concentration (wt.%) at a fixed frequency of 30 GHz.

The frequency dependencies of dielectric permittivity and dielectric losses, and the concentration dependence of complex dielectric permittivity, are presented in Figure 4. Both dielectric permittivity and dielectric losses exhibit a strong decrease with frequency for all investigated samples. Such behavior is in good agreement with Jonsher’s universal power law [37]. According to this law, both dielectric permittivity and dielectric losses decrease with frequency in a power law fashion [37]. The observed values of dielectric permittivity (ε′ ≈ 10–30) and dielectric losses (ε″ ≈ 1–25) are high enough to suggest that these composites are suitable for electromagnetic shielding applications [36,38]. Indeed, it was demonstrated that a 1 mm sample layer, prepared from materials with ε′ > 15 and ε″ > 8, exhibited absorption as great as 50% [36].

Dielectric losses are higher for CIP-containing composites than for carbon structures (Figure 4b,c), with the highest losses observed in composites containing 20 wt.% CIP. In contrast, the concentration dependence of dielectric permittivity is less pronounced, with only the composite containing 35 wt.% CIP showing a slightly lower permittivity than the other structures. The influence of CIP on the dielectric properties of the investigated composites is less marked than in polymer composites with the same filler [6,7,8,9,10], likely due to the screening effect of unbound electrons in the carbon materials [38]. The frequency-dependent dielectric properties observed in the microwave frequency range for the materials studied are attributed to electron transport within the composite, which is consistent with the behavior reported for other carbon-based materials investigated in microwaves [28].

The scattering parameters of a layer of composite studied in vacuum can be calculated using the following equations [36]:

(3) S11=−jkzk2z2−1sin⁡k2zτ2k2zkzcosk2zτ+jk2zkz2−1sin⁡k2zτ,

(4) S21=2k2zkz2k2zkzcosk2zτ+jk2zkz2−1sin⁡k2zτ,

where k_z_ = 2π/λ and k_2z_ = 2πε^0.5^/λ are the wavenumbers in vacuum and the material medium, respectively, and τ is the layer thickness. The reflectivity, transmission and absorption of the layer can be calculated as R = |S_11_|^2^, T = |S_21_|^2^ and A = 1 − R − T, respectively.

**Figure 5:**
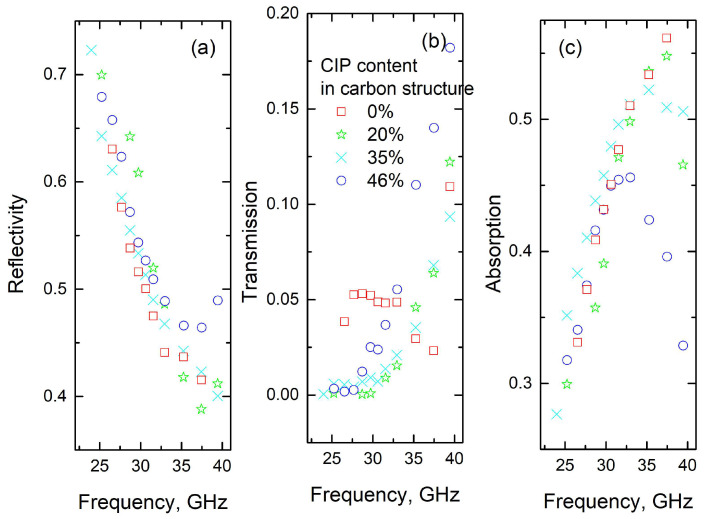
Microwave properties (reflectivity R (**a**), transmission T (**b**), absorption A (**c**)) of carbon structures as a function of frequency at different CIP concentrations.

The microwave absorption properties of the composites, calculated using Equations (3) and (4), along with the complex permittivities measured (Figure 4) for 2 mm-thick plates, are presented in Figure 5. The electromagnetic behavior differs below and above 30 GHz. Below 30 GHz, absorption increases strongly with frequency, but is nearly independent of particle concentration. At frequencies higher than 30 GHz, absorption becomes dependent on the concentration of magnetic inclusions, and for a concentration of 46%, decreases with frequency. In this frequency range, adsorption improves with low CIP concentrations (around 20%), while decreasing at higher filler concentrations (above 35%). The best absorption properties are observed in structures containing 20 wt.% inclusions, with adsorption values reaching 52% at 35 GHz (for both experiments and calculations for 2 mm-thick slabs).

Previous studies [6,7,8,9,10] have reported significant improvements in microwave adsorption through the addition of CIP particles to polymer composites. In the studies cited, magnetic particles and various carbon structures were incorporated into a polymer matrix, resulting in significant improvement in the composites’ macroscopic conductivity and dielectric properties. In contrast, the composites studied here feature magnetic particles embedded in an electrically conductive carbon matrix. As a result, electromagnetic properties in the microwave range are primarily dominated by the total conductivity of the structure, which in turn are influenced by the density of the composite. Moreover, the electrical conductivity of the structures are dominated by their density (Figure 3). This explains why a higher concentration (46 wt.%) of magnetic particles tends to reduce absorption in these composites.

The λ/4 geometry, commonly known as the Salisbury screen, can be also used in the microwave frequency range for shielding applications [39]. In this configuration, a layer of material is placed on a back reflector, with the incident wave reflecting off both the first edge of the layer and the back reflector. These two reflected signals interfere. If the phase difference is π, the interference causes the total reflected wave to be reduced to 0%. The system can be modeled simply in the case of a lossless material (ε′′ = 0). A simple resonant equation can be used to estimate the required layer thickness, which satisfies the condition of a totally reflection-free structure. However, for materials with non-zero dielectric losses, the propagation of electromagnetic radiation is more complicated, since the wave reflected by the back reflector is subject to an additional phase angle rotation. Appropriately, the fixed-frequency resonance condition can only be met for specific values of dielectric permittivity, dielectric losses and thickness.

For the system with a back reflector, the scattering parameter S_11_ is as follows [40]:

(5) $$S_{11}=-\frac{k_{z}\left(\exp\left\{2i\tau k_{2z}\right\}-1\right)+k_{2z}\left(\exp\left\{2i\tau k_{2z}\right\}+1\right)}{k_{z}\left(-\exp\left\{2i\tau k_{2z}\right\}+1\right)+k_{2z}\left(\exp\left\{2i\tau k_{2z}\right\}+1\right)}.$$

**Figure 6:**
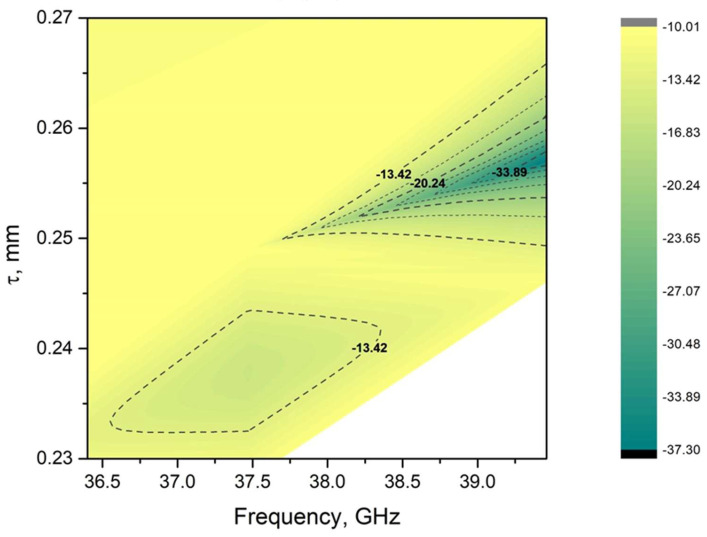
Parameter S11 of the sample layer containing 46 wt.% CIP as a function of frequency and thickness.

As in Equations (3) and (4), k_z_ and k_2z_ are the wavenumbers, ε is the permittivity of the composite and τ is the thickness. The dependence of S_11_ (in dB) on thickness and frequency is presented in Figure 6.

The materials presented show promise for Salisbury screen applications [40]. In particular, the S_11_ value of the sample containing 46 wt.% CIP is less than −33 dB, which corresponds to more than 99.99% of the incident irradiation. The frequency and layer thickness ranges are narrow due to the resonant nature of the Salisbury screen. Specifically, a very small change in thickness from 0.25 mm to 0.24 mm (such a change is experimentally available) corresponds to an increase in S_11_ from <−34 dB to >−13 dB. However, this absorption range can be modified, as it has previously been shown that a small change in temperature (i.e., 5–7 K) can shift the position of the peak shielding efficiency of Salisbury screens (SER = |20log10(S_11_)|) [40]. As the CIP concentration decreases, the system gradually moves out of resonance and S_11_ increases. Finally, for the sample without CIP, the −20 dB level is impossible to achieve.

## 4. Conclusions

Carbon–CIP composite samples were prepared by 3D printing tannin-based photosensitive resin and CIP microparticles, followed by heat treatment of the resulting preforms. The electrical conductivities of the investigated structures were about several S/cm in the radio frequency range (20 Hz–1 MHz), consistent with typical values for glassy carbon. The temperature dependence of the DC conductivity followed Arrhenius’ law, with activation energies of several tens of meV, characteristic of electron transport through various defects in the carbon matrix. The addition of CIP increased the dielectric losses of the composite materials, while the dielectric permittivity remained largely unaffected by CIP concentration. Notably, at 35 GHz and with 20 wt.% CIP, electromagnetic absorption reached 52%. Furthermore, carbon composites with the highest CIP concentration exhibited interesting resonance states, demonstrating significant potential for Salisbury screen applications. Given their customizable design, made possible by laser stereolithography, these carbon–CIP structures have great potential for electromagnetic shielding applications.

## Figures and Tables

**Table 1:** Characteristics of carbon–CIP composites.

Initial CIP Concentration, Φ_i_ (wt.%)	Final CIP Concentration, Φ_f_ (wt.%) *	Final CIP Concentration, Φ_v_ (vol.%) **	Bulk Density, ρ_b_ (g/cm^3^)
0	0	0	0.42
5	20	1	0.44
10	35	2	0.51
15	46	4	0.67

* Calculated as Φ_f_ = Φ_i_/[(1 − Y_c_)∙Φ_i_ + Y_C_] where Y_C_ = 20.8% is the carbon yield of the acrylate-tannin resin upon pyrolysis at 900 °C. ** Φ_V_ = Φ_f_∙ρ_b_/7.86, where 7.86 g/cm^3^ is the bulk density of carbonyl iron powder.

**Table 2:** Parameters for approximating Arrhenius’ law approximation.

CIP Concentration, wt.%	Temperature Region, K	σ_0_, S/cm	E/k, K (meV)
0	T < 155	7.2	137 (11.8)
	T > 155	9.6	184 (15.8)
20	T < 186	2.27	302.7 (26)
	T < 186	1.02	152.1 (13.1)
35	T < 227	11.98	131.9 (11.3)
	T > 259	11.98	186.3 (13)
46	T < 220	1.79	275.6 (23.7)
	T > 250	1.15	147.2 (12.6)

## Data Availability

The original contributions presented in the study are included in the article, further inquires can be directed to the corresponding author.
